# Gut microbiota and osteoarthritis: epidemiology, mechanistic analysis, and new horizons for pharmacological interventions

**DOI:** 10.3389/fcimb.2025.1605860

**Published:** 2025-07-16

**Authors:** Nianyi Sun, Yinuo Zhao, Anren Zhang, Yu He

**Affiliations:** ^1^ Department of Rehabilitation, Shanghai Fourth People’s Hospital, School of Medicine, Tongji University, Shanghai, China; ^2^ School of Medicine, Tongji University, Shanghai, China; ^3^ Department of Rehabilitation, Shengjing Hospital of China Medical University, Shenyang, China

**Keywords:** osteoarthritis, gut microbiota, dysbiosis, inflammation, metabolites, cartilage

## Abstract

Emerging evidence suggests that gut microbiota dysbiosis is associated with the onset and progression of osteoarthritis (OA). While OA was traditionally considered a localized degenerative joint condition, it is now increasingly viewed as a systemic disorder involving low-grade inflammation and metabolic imbalance. This review synthesizes current findings on the gut–joint axis and presents a structured overview of how alterations in microbial communities may relate to phenotypic variability in OA. Observational studies have identified correlations between gut dysbiosis and factors such as obesity and hyperuricemia, which are themselves linked to increased intestinal permeability, elevated circulating lipopolysaccharide levels, and reduced production of short-chain fatty acids. These features may contribute to immune dysregulation and tissue degeneration, although definitive causal mechanisms remain unconfirmed. Some reports have also detected microbial DNA in joint tissues, raising the possibility of microbial translocation and its potential role in local inflammatory processes. In light of these associations, we review several microbiota-directed interventions, including probiotics, dietary supplements, Traditional Chinese Medicine, and biomaterial-based approaches. Although preliminary studies suggest these strategies may influence systemic inflammation and joint health, most evidence is derived from preclinical models or small-scale clinical trials. Causality has not yet been firmly established, and further validation in larger, well-controlled studies is needed. By integrating current mechanistic insights with emerging therapeutic directions, this review highlights the potential relevance of the gut–joint connection in OA and underscores the importance of continued research toward microbiota-informed, individualized approaches to disease understanding and management.

## 1 Introduction

Osteoarthritis (OA) is one of the most common degenerative joint diseases, marked by cartilage degradation, osteophyte formation, synovial inflammation, and progressive pain and functional impairment (van den Bosch, 2020; Duong et al., 2023). With global life expectancy on the rise, OA has become a leading cause of disability and healthcare burden, particularly among ageing populations. This trend is exacerbated by the increasing prevalence of metabolic conditions such as obesity and insulin resistance, which are closely linked to both the onset and progression of OA (Steinmetz et al., 2023; Tang et al., 2025). Although it was traditionally regarded as a localized process driven primarily by mechanical wear and tear with minimal inflammatory involvement, current understanding has shifted to recognize OA as a multifactorial disease in which systemic processes play a crucial part. Metabolic dysregulation and chronic, low-grade inflammation are now considered major contributors to cartilage degradation, heightened pain sensitivity, and disease progression (Steves et al., 2016; Ryu et al., 2020; Wei et al., 2023; Yang et al., 2024).

The gut microbiota constitutes a diverse and dynamic microbial ecosystem that influences host physiology through immune, metabolic, and neuroendocrine pathways. Recent advances in high-throughput sequencing and integrative omics technologies have revealed extensive associations between gut dysbiosis and chronic inflammatory conditions, including cardiovascular diseases, diabetes, and OA (Foley et al., 2019; Seely et al., 2021; Talmor-Barkan et al., 2022; Jang et al., 2024; Wang et al., 2025). Notably, alterations in microbial composition—often observed in individuals with obesity, hyperuricemia, or metabolic syndrome—may compromise intestinal barrier integrity, increase endotoxin translocation, and activate immune cascades that contribute to joint inflammation and cartilage degradation (Pushpakumar et al., 2021; Hou et al., 2022; Graham and Xavier, 2023; Byndloss et al., 2024).

Support for the gut–joint axis has emerged from epidemiological and clinical studies. For example, specific microbial metabolites derived from tryptophan have been linked to increased joint pain, while reduced microbial diversity has been associated with greater OA severity in certain populations (Pedersini et al., 2020; Li et al., 2021). Moreover, the detection of microbial DNA in synovial fluid and cartilage suggests a possible translocation of gut microbes or microbial components to joint tissues, directly contributing to local immune activation and structural damage (He and Chen, 2022; Basak et al., 2023).

Mechanistic studies have identified several key microbial-derived molecules—such as lipopolysaccharide (LPS), short-chain fatty acids (SCFAs), and altered amino acid metabolites—that influence immune responses, chondrocyte homeostasis, and nociceptive signaling (Rahman et al., 2023; Karim, 2024). These findings underscore the importance of the intestinal mucosal barrier as a regulatory interface in OA, particularly among metabolically vulnerable individuals.

In light of these insights, the gut microbiota has become a promising target for disease-modifying interventions. Beyond conventional OA treatments focused on symptom control and surgical repair (Katz et al., 2021), emerging strategies such as probiotics, prebiotics, dietary modulation, and functional supplements are being investigated for their capacity to reshape host–microbiome interactions. Traditional Chinese Medicine (TCM), including herbal formulations and external therapies like acupuncture and moxibustion, has also demonstrated potential in modulating gut microbial balance and attenuating joint pathology via immunometabolic pathways (Li et al., 2024).

This review synthesizes recent advances in understanding the gut microbiota’s role in OA pathogenesis. We discuss epidemiological and clinical evidence, characterize microbial compositional and metabolic features associated with OA, elucidate key molecular mechanisms, and evaluate microbiota-targeted therapies, including TCM-based approaches. By integrating findings across human, animal, and cellular models, this review aims to clarify the gut–joint axis and explore its translational potential in OA management and prevention.

## 2 Epidemiological studies on gut microbiota and osteoarthritis

**Figure 1:**
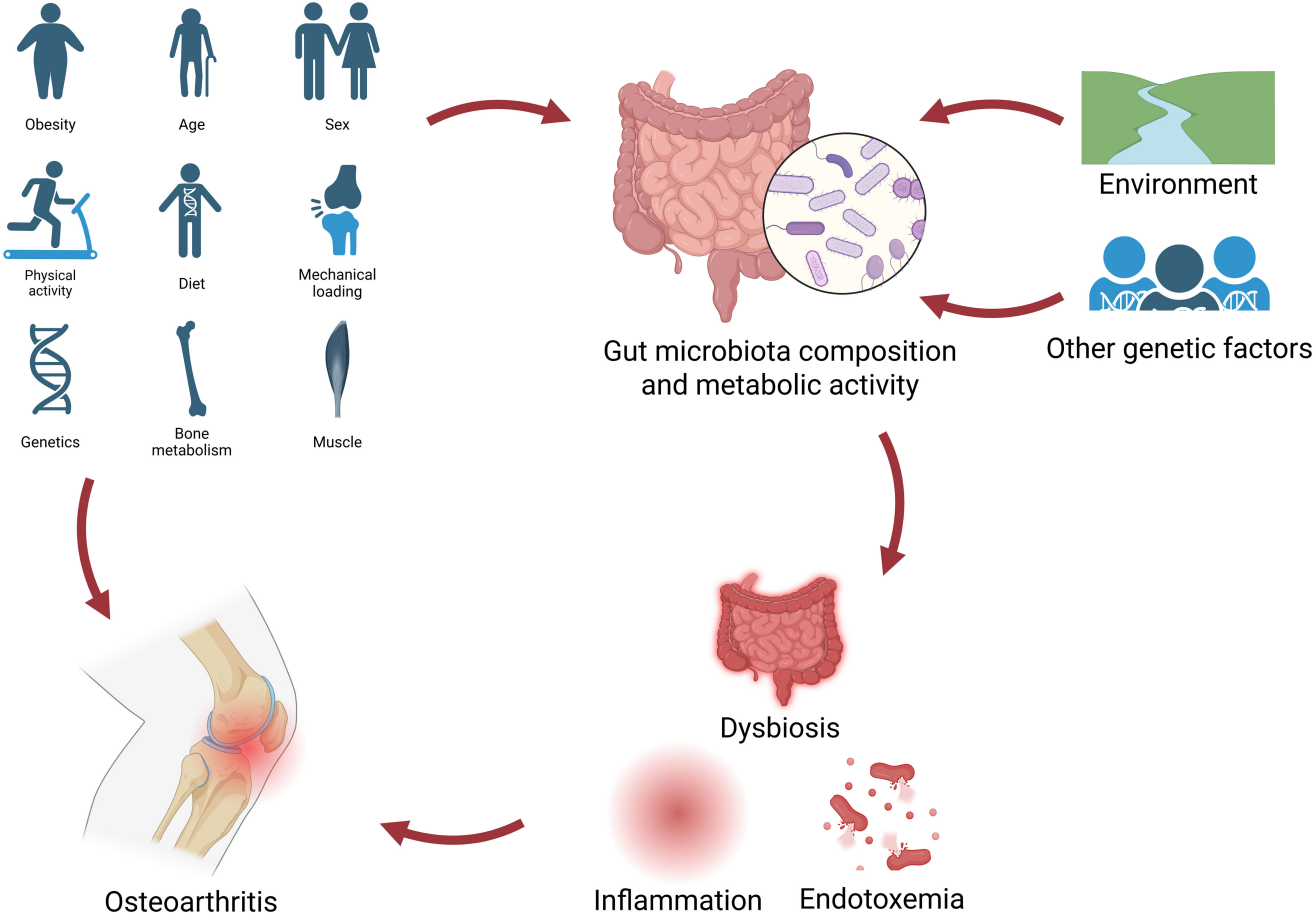
Interactions between gut microbiota and risk factors of OA. Risk factors of OA exhibit a dual pathogenic mechanism, in that they can directly trigger OA and indirectly accelerate its progression by disrupting gut microbial homeostasis. The gut microbiota is modulated not only by the host genetic background but also significantly responds to environmental exposures, thereby indirectly influencing OA pathogenesis.

Traditional epidemiological studies of OA have primarily focused on conventional risk factors, including obesity, ageing, and joint mechanical loading (Bennell et al., 2022; Messier et al., 2022; Ferrari et al., 2024). However, with the rapid advancement of high-throughput single-cell transcriptomics and metabolomics technologies, researchers have increasingly recognized the potentially significant role of gut microbiota in systemic inflammation and metabolic disturbances (**Figure 1**) (Han et al., 2021; Muñoz et al., 2024; Delzenne et al., 2025; Shen et al., 2025). Multiple large-scale population-based and cohort studies have further uncovered a potential association between gut microbiota and OA initiation and progression (**Table 1**) (Boer et al., 2019; Wei et al., 2021; Wei et al., 2022; Binvignat et al., 2023; Wei et al., 2023a). From the association between disturbed tryptophan metabolism and hand OA related pain to the connections linking intestinal dysbiosis with hyperuricemia and joint pain, these findings collectively offer supporting evidence for the hypothesis of a “gut–joint axis”.

**Table 1:** Overview of major epidemiological/cohort studies.

Source cohort research title	Sample size	Experimental group size	Control group and its conditions	Measurement	Findings	Conclusion	Reference
Xiangya osteoarthritis study (Hand OA)	1388	**72**	1316; Significantly higher abundance of *Roseburia*; Markedly lower abundance of pro-inflammatory genera	Bacterial 16S rRNA gene	The abundance of pro-inflammatory bacteria *Bilophila* and *Desulfovibrio* increased, while the butyrate-producing bacterium *Roseburia* decreased	Suggests a close association between dysbiosis, low-grade inflammation, and hand OA	(Wei et al., 2021)
DIGICOD cohort	416	141	275; A more comprehensive tryptophan metabolic profile; lower activity of inflammatory pathways; and milder clinical symptoms	Quantification of tryptophan metabolites	Molecules such as indole-3-aldehyde are significantly associated with the severity of erosive hand OA.	Suggests a close association between tryptophan derivatives and joint pain and inflammation	(Binvignat et al., 2023)
Independent cohort study of hand OA	1359, 142	discovery cohort: 70; validation cohort: 71	discovery cohort: 1289; validation cohort: 71; Enhanced amino acid metabolism pathways and cofactor metabolism	Quantification of tryptophan metabolites	Several tryptophan metabolites were elevated, while indole-3-lactic acid and skatole decreased.	Further validates the association between tryptophan imbalance and joint pain	(Wei et al., 2023a)
Xiangya osteoarthritis study (Hyperuricemia)	1392, 480	discovery cohort: 239; validation cohort: 240	discovery cohort: 1153; validation cohort: 240; Gut microbiota of individuals with healthy serum uric acid levels exhibits greater diversity and stability	Bacterial 16S rRNA gene Functional pathway analysis	In individuals with hyperuricemia, gut diversity decreased, amino acid/nucleotide pathways were upregulated, and the beneficial bacterium *Coprococcus* was reduced.	Suggests that individuals with hyperuricemia are more susceptible to damage in the microbiome-metabolism-arthritis inflammation pathway	(Wei et al., 2022)
Rotterdam Study Lifelines-DEEP	1427, 867	discovery cohort: 285; validation cohort: 197	discovery cohort: 1142; validation cohort: 670; High microbial diversity and low abundance of streptococci	Bacterial 16S rRNA gene	Joint pain is associated with an increase in the abundance of *Streptococcus* species and is significantly correlated with local inflammation.	Hypothesizes that the "microbial endotoxin-macrophage" axis exacerbates OA pain	(Boer et al., 2019)

While these studies have advanced our understanding of the potential microbiome–articular connection, it is important to recognize their methodological constraints. Although cross-sectional studies have yielded important insights supporting the hypothesis of a microbiome–articular axis, their inherent limitations prevent the establishment of definitive causal relationships between microbial alterations and OA pathogenesis, thus necessitating cautious interpretation of the findings (Wei et al., 2021). In addition, notable methodological heterogeneity exists—for example, Wei et al. employed a metagenomic sequencing approach, whereas Boer et al. used 16S rRNA gene sequencing—making direct comparisons between studies challenging (Boer et al., 2019; Wei et al., 2023a). To address these issues, future research should adopt multidimensional, longitudinal designs and standardized sequencing methodologies to strengthen the evidence base for microbiome-targeted therapeutic strategies in OA.

### 2.1 Gut microbiota and hand osteoarthritis

Several independent cohort studies focusing on hand OA have consistently emphasized the critical metabolic pathway involving tryptophan derivatives. The DIGICOD cohort, comprising 416 patients with hand OA, systematically measured 20 distinct tryptophan metabolites and identified specific molecules such as indole-3-aldehyde as significantly correlated with the severity of erosive hand OA (Binvignat et al., 2023). Another investigation involving two independent populations similarly reported elevated levels of multiple tryptophan metabolites in symptomatic hand OA patients, accompanied by a marked reduction in indole-3-lactic acid and skatole (Wei et al., 2023a). Despite differences in study populations and experimental designs, both studies consistently reinforced a close association between disrupted tryptophan metabolism and joint pain or erosion severity. Given that tryptophan metabolism relies heavily on gut microbiota to produce numerous bioactive metabolites, gut dysbiosis may be associated with inflammation and pain phenotypes in hand OA (Agus et al., 2021).

The Xiangya Osteoarthritis Study, conducted on the same community-based population, focused on the relationship between “symptomatic OA” and the macro composition of the gut microbiota (Wei et al., 2021). In total, 1,388 participants were enrolled, and the analysis revealed significant alterations in gut microbial β-diversity among individuals diagnosed with symptomatic hand OA based on clinical symptoms and radiographic findings. Specifically, an increased abundance of potentially pro-inflammatory and hydrogen sulfide-producing bacteria, such as *Bilophila* and *Desulfovibrio*, was observed alongside a notable reduction in typical butyrate-producing beneficial bacteria, exemplified by *Roseburia*. This microbial pattern of “increased pro-inflammatory bacteria and decreased beneficial bacteria” aligns closely with the previously discussed concept of microbial dysbiosis driving low-grade inflammation suggested by tryptophan metabolism studies, and further suggests a possible link between systemic inflammatory signals and local pathological changes in hand OA (Mulrooney et al., 2021).

### 2.2 Gut microbiota and knee osteoarthritis

Large-scale cohort studies have similarly demonstrated t a potential association between gut microbiota and knee OA and its associated pain phenotypes. The Rotterdam Study and the Lifelines-DEEP study, covering extensive populations of European adults, employed 16S sequencing and absolute quantitative analyses, revealing a significant correlation between joint pain and increased abundance of the genus Streptococcus (Boer et al., 2019). Subsequent analyses further suggested that this association is closely related to local joint inflammation, proposing that macrophage-mediated inflammation, possibly related to microbial endotoxins, may be associated with joint pain progression.Within the broader spectrum of metabolic factors, hyperuricemia represents another significant research focus (Taskina et al., 2021). Based on 1392 participants from the Xiangya Osteoarthritis Study, and subsequently replicated in another validation cohort, it was observed that individuals with hyperuricemia exhibited a significantly reduced gut microbial diversity, alongside enhanced functional pathways related to amino acid and nucleotide metabolism, and a decreased abundance of beneficial bacterial genera, including *Coprococcus* (Wei et al., 2022). Such patterns of gut microbial dysbiosis, frequently associated with obesity or gout, further suggest that OA individuals with concurrent hyperuricemia may be more susceptible to patterns observed along the microbiota–metabolism–joint inflammation axis (Wang et al., 2024).

Gut microbial dysbiosis at both compositional and functional levels, including disturbances in amino acid metabolism, enrichment of pro-inflammatory bacterial genera, and depletion of critical beneficial bacteria, may contribute to the initiation or exacerbation of OA clinical manifestations (Biver et al., 2019). Although existing epidemiological studies have predominantly adopted cross-sectional designs, making it challenging to determine causality between microbiota alterations and OA, these studies clearly highlight a close relationship between gut microbiota and OA (Wei et al., 2023b). In the future, if these results can be further validated in larger prospective or interventional studies, it will help to clarify the potential value of gut microbiome intervention in the prevention and treatment of OA.

### 2.3 Other studies

In studies exploring the relationship between gut microbiota and OA, certain findings have not demonstrated significant differences in microbial diversity or composition. A metagenomic sequencing analysis involving 93 dogs with naturally occurring OA reported no significant differences in microbial α and β diversity between the pain group and healthy controls (Stevens et al., 2024). Similarly, another cross-sectional study conducted in an obese human population observed significantly elevated serum LPS levels in the OA group but found no substantial difference in microbial composition compared to controls (Loeser et al., 2022). The negative results do not negate the importance of intestinal microbiota in OA, but provide some suggestions for the subsequent work in terms of research objects, sampling scale, analysis methods and selection of microbiota function indicators.

Taken together, these epidemiological findings lay the groundwork for exploring underlying biological mechanisms through which gut microbiota may contribute to OA pathology.

## 3 Gut microbiota and metabolic characteristics in osteoarthritis

### 3.1 Microbial DNA in cartilage

Previous studies tended to focus on microbial dysregulation in intestinal samples (Hao et al., 2021; Sánchez Romero et al., 2021). The research on whether there is microbial DNA in articular cartilage has made this issue a new hot topic (Dunn et al., 2020; Izda et al., 2023). After inoculating germ-free mice with normal gut microbes, microbial DNA was detectable within articular cartilage within only 48 hours. Such rapid deposition indicates cross-tissue microbial translocation from the gut to joints, and further implies that microbes or their genomic components raises the possibility that microbes or their genomic components are associated with local inflammation. Moreover, when mice experienced obesity, high-fat diet feeding, or osteoarthritis induced by anterior cruciate ligament or meniscal injury, cartilage microbial DNA exhibited synchronous alterations with the gut microbiota, suggesting a close association between systemic metabolic states and joint pathology (Izda et al., 2023). Another study using 16S sequencing in human knee and hip cartilage samples identified significantly decreased α-diversity in OA cartilage, along with relative enrichment of *Gram-negative bacteria*, along with the up-regulation of genes related to inflammation and metabolic pathways such as LPS synthesis and phosphatidylinositol signaling (Dunn et al., 2020). These findings support the concept that joints might not be completely sterile, and microbial DNA translocation across mucosal barriers into the joint may amplify local inflammatory signaling, thereby accelerating cartilage degeneration.

### 3.2 Endemic osteoarthritis: Kashin-Beck disease

Kashin-Beck disease (KBD), an endemic osteoarthritis subtype prevalent in specific regions of China, has been investigated through 16S rRNA gene sequencing and serum metabolomics analyses comparing patients with healthy controls (Wang et al., 2021). The results demonstrated increased abundances of bacterial phyla, including *Fusobacteria* and *Bacteroidetes*, along with significant enrichment of bacterial genera such as *Alloprevotella*, *Megamonas*, and *Escherichia_Shigella* in the KBD population. Furthermore, widespread disturbances in lipid metabolism pathways, particularly involving unsaturated fatty acids and glycerophospholipids, were identified among KBD subjects. These metabolic abnormalities were significantly correlated with changes in specific microbial genera, providing typical evidence that “gut microbial dysbiosis coupled with host metabolic disturbance” may contribute to or be associated with osteochondral pathology. Given that nutritional and environmental toxin-related factors may also be involved in KBD pathogenesis, findings further illustrate that microbial-host interactions remain a critical pathogenic pathway even in relatively rare or distinct OA subtypes.

### 3.3 Obesity associated osteoarthritis

In addition to geographical factors, obesity is also recognized as an important trigger for the high incidence of OA (Elmaleh-Sachs et al., 2023; Delzenne et al., 2025). A study employing fecal untargeted metabolomics and 16S sequencing in obese patients with concurrent hand and knee OA revealed significant accumulation of dipeptides and tripeptides, alongside disrupted metabolic pathways involving multiple amino acids and lipids, as well as abnormal levels of microbial-derived metabolites such as propionate and indoles (Rushing et al., 2021). Imbalanced proteolysis and microbial-metabolic disturbances are frequently associated with increased intestinal permeability and chronic low-grade inflammation, which may be associated with more severe joint degeneration and pain. Furthermore, regression modeling in this study demonstrated that these metabolic characteristics, in combination with microbial abundance, could be utilized to discriminate phenotypes among obese OA populations. Within the complex metabolic context of obesity, gut microbial modulation of protein, amino acid, and lipid metabolism is associated with variations in systemic and joint inflammation levels (Sampath et al., 2023; Binvignat et al., 2024; Li et al., 2024). These findings also provide potential biomarkers and novel perspectives for future microbiota-targeted therapeutic interventions in obese OA populations.

### 3.4 Post traumatic osteoarthritis and exercise intervention

Post traumatic osteoarthritis (PTOA), a degenerative condition triggered by injuries to joint structures such as ligaments or menisci, differs etiologically from primary OA (Jeon et al., 2017; Kraus and Hsueh, 2024). A study demonstrated that gut microbiota also plays a significant role in PTOA models (Hao et al., 2022). Rats undergoing ligament and meniscal injury surgery commonly exhibited a pathological microbiota shift characterized by increased abundance of *Fusobacteria* and reduced proportions of potentially beneficial bacteria. Importantly, eight weeks of treadmill exercise partially reversed these microbial alterations, helping to preserve the structural integrity of cartilage and subchondral bone while concurrently reducing systemic inflammatory markers. These findings further support the beneficial regulatory effects of exercise on gut microbiota composition, supporting the potential relevance of the “gut–joint” axis theory across OA types with different etiologies and phenotypes. By maintaining or restoring a healthy microbiota, exercise may confer benefits to PTOA patients, protecting joints not only at the mechanical level but also through immunological and metabolic mechanisms (Gao et al., 2024; Hawley et al., 2025).

**Figure 2:**
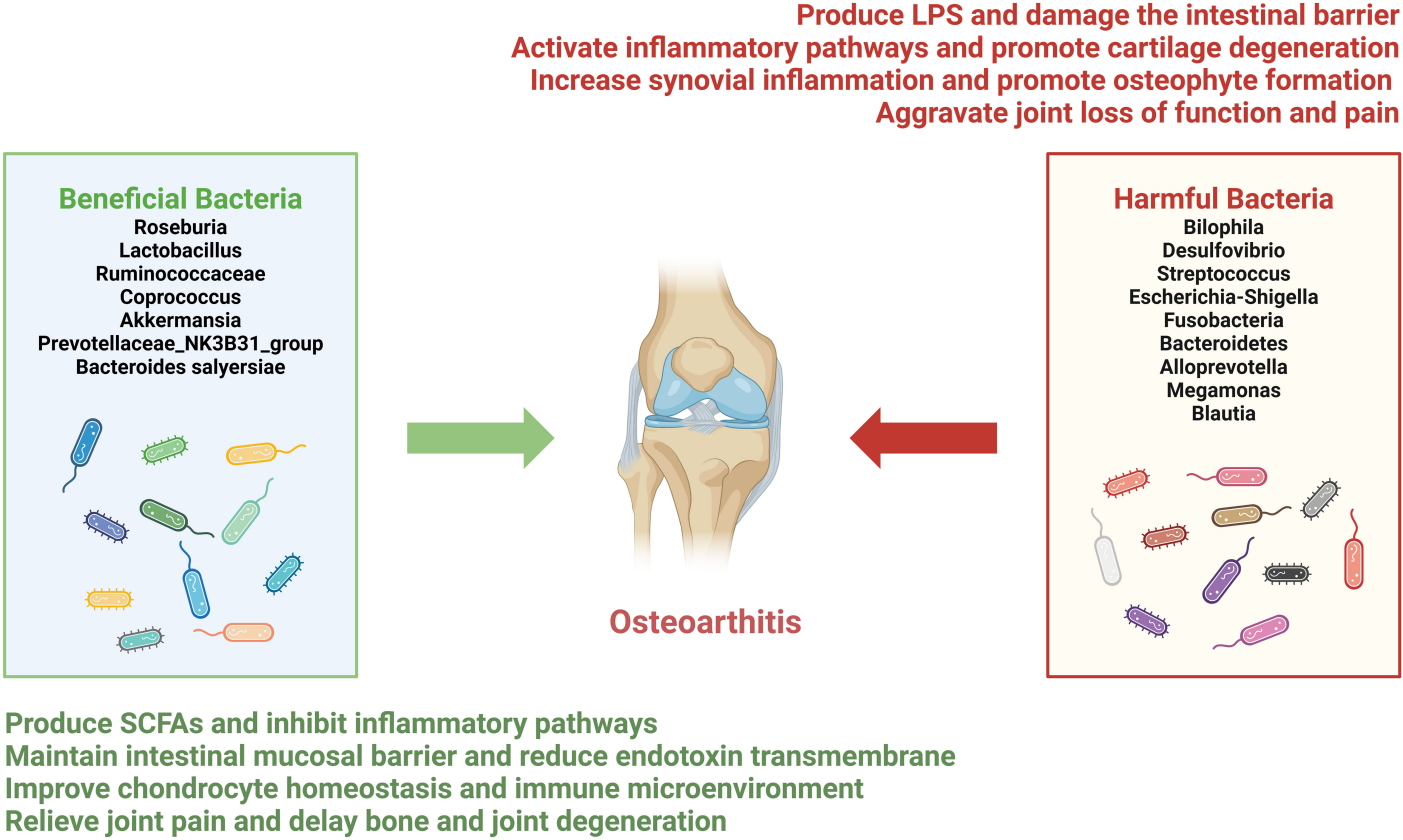
Dual regulatory role of gut microbiota in osteoarthritis. Gut microbiota associated with OA are categorized into beneficial bacteria (left panel) and harmful bacteria (right panel) groups. Beneficial bacteria, including *Roseburia*, *Lactobacillus*, *Ruminococcaceae*, *Coprococcus*, *Akkermansia*, *Prevotellaceae_NK3B31_group*, and *Bacteroides salyersiae*, contribute to reduced cartilage degradation and inflammation primarily through the production of short-chain fatty acids, restoration of intestinal mucosal barrier integrity, and modulation of immune homeostasis. Conversely, harmful bacteria, such as *Bilophila*, *Desulfovibrio*, *Streptococcus*, *Escherichia-Shigella*, *Fusobacteria*, *Bacteroidetes*, *Alloprevotella*, *Megamonas*, and *Blautia*, when increased in abundance, are frequently associated with elevated levels of LPS and other pathogenic metabolites, leading to exacerbation of synovial inflammation, pain, and cartilage degeneration.

Across diverse OA subtypes and pathological contexts, the critical interplay between microbial dysbiosis and host metabolic disturbances consistently emerges as a central theme (Chen et al., 2022; Fábio, 2022; Gaspar et al., 2024). Whether exogenous microbial DNA crosses the intestinal mucosa and deposits in the joint, or the double disturbance of microbiota and metabolism brought by high fat diet and environmental toxins, all point to the key position of “microbiota–metabolism–inflammation” in promoting the degeneration of articular cartilage (**Figure 2**).

With continuous advances in analytical technologies and omics approaches, researchers are now able to achieve increasingly refined and multidimensional characterization of both the joint microenvironment and gut ecosystem, thereby providing new opportunities for further dissecting OA pathogenesis and exploring novel microbiota-targeted intervention strategies (Rozera et al., 2025). These findings underscore the complex and multi-level influence of gut microbiota across OA phenotypes. This has prompted growing interest in uncovering the specific microbial and molecular pathways that may mediate such effects.

## 4 Mechanisms underlying gut microbiota effects on osteoarthritis

### 4.1 Endotoxin and low grade inflammation

**Figure 3:**
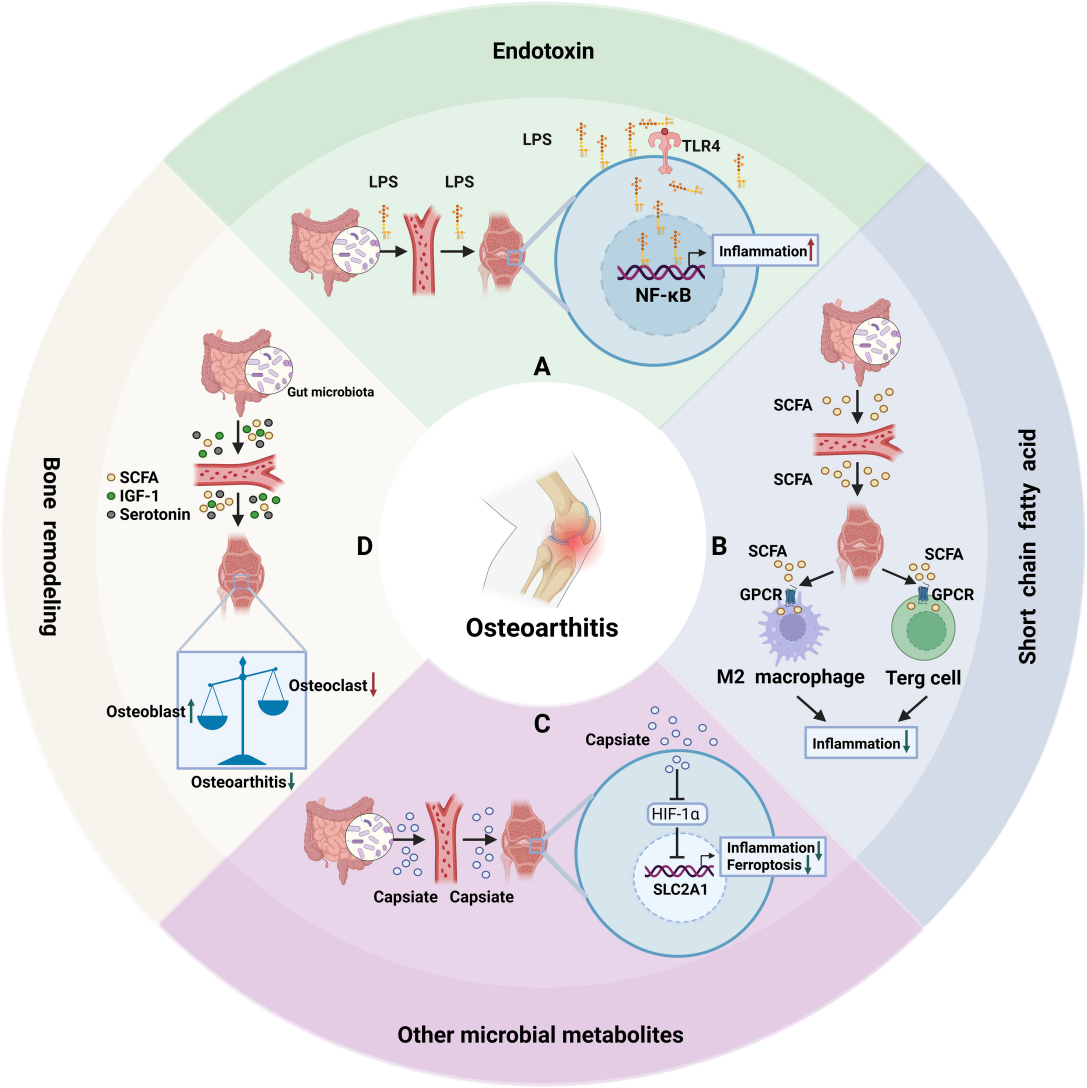
Mechanisms underlying gut microbiota effects on OA. **(A)** Under conditions such as obesity, metabolic syndrome, or compromised intestinal mucosal barriers, elevated levels of LPS translocate from the gut into systemic circulation. Subsequently, LPS amplifies inflammatory responses in synoviocytes and chondrocytes through activation of signaling pathways including TLR4/NF-κB, leading to joint pain and tissue degeneration. **(B)** SCFAs produced via microbial fermentation in the gut, modulate immune homeostasis through GPCRs. Specifically, SCFAs promote the differentiation and activity of regulatory T cells (Tregs) and M2 macrophages, thereby suppressing inflammation and preserving chondrocyte homeostasis. **(C)** The gut-derived metabolite capsiate suppresses HIF-1α expression and upregulates SLC2A1 to alleviate chondrocyte ferroptosis and inflammation. **(D)** SCFAs, IGF-1, serotonin and other microbial metabolites influence the balance between osteoblast and osteoclast activities, thereby ameliorating abnormal bone remodeling in OA.

Individuals with obesity and metabolic syndrome often exhibit impaired intestinal mucosal barrier function, allowing LPS translocation from the intestinal lumen into systemic circulation, thereby exacerbating systemic low-grade inflammation and worsening the course of OA (Liu et al., 2023; Li et al., 2024). A “two-hit” model proposed by a previous study emphasized that elevated serum LPS levels, in the context of pre-existing joint injury, amplify inflammatory responses in synovial and cartilage cells through pathways such as TLR4/NF-κB, subsequently aggravating OA severity (**Figure 3A**) (Huang and Kraus, 2015). In this model, the first “hit” involves systemic metabolic dysfunction—most notably, elevated circulating LPS resulting from compromised gut barrier integrity—which primes the innate immune system to mount an inflammatory response. The second “hit” consists of joint injury or mechanical stress, which further stimulates inflammation in this already sensitized immune environment. Acting in concert, these two factors accelerate joint degeneration and drive OA progression. Concurrent studies have also indicated that increased LPS burden is common not only in obese OA patients (Huang et al., 2016). Analysis of serum and synovial fluid samples from knee OA patients revealed significant associations of LPS and LPS-binding protein levels with joint pain, osteophyte formation, and local macrophage activation. These findings suggest that both systemic and intra-articular LPS exposure can be linked to joint structural abnormalities and inflammatory symptoms (Bonato et al., 2021; Franco-Trepat et al., 2021). Furthermore, microbial transplantation experiments indicated that gut microbiota from metabolically impaired individuals could induce higher inflammation levels and more severe degenerative joint changes in recipient mice, often associated with elevated transmembrane absorption of LPS (Huang et al., 2020). One study revealed sex-specific susceptibility to microbiota alterations, showing that male mice were more prone to developing severe OA, potentially linked to stronger pro-inflammatory signaling (Schlupp et al., 2023). Collectively, these findings indicate that LPS-mediated subclinical inflammation occupies a central role in OA progression in the context of obesity, sex differences, or metabolic syndrome.

### 4.2 Short chain fatty acids and immune regulation

In contrast to the pro-inflammatory effects mediated by LPS, SCFAs are generally considered immunoprotective factors derived from gut microbiota (Kim, 2021; Liu et al., 2024). A study focusing on knee osteoarthritis demonstrated that administration of live *Lactobacillus* (*strain LA-1*) promoted the production of SCFAs such as butyrate, enhanced cartilage autophagy, and reduced necroptosis and synovial inflammation (Cho et al., 2022). Another study similarly confirmed that supplementation with Lactobacillus rhamnosus or butyrate ameliorated joint destruction and inflammation, highlighting the significant role of SCFAs in suppressing inflammatory pathways in chondrocytes and regulating cellular autophagy and apoptosis (Jhun et al., 2021). From a broader perspective on microbial-nutritional interactions, research has shown that strains such as *Bacteroides salyersiae* efficiently degrade chondroitin sulfate, releasing substantial amounts of SCFAs and chondroitin sulfate oligosaccharides (Wang et al., 2024). These metabolites not only support the growth requirements of commensal bacteria but may also provide novel anti-inflammatory or reparative factors beneficial to the host. Collectively, SCFAs mediate immune homeostasis through G protein-coupled receptors (GPCR) or epigenetic dependent modulation of regulatory T cells (Tregs) and M2 macrophages, suggesting that microbiota serve as a critical bridge linking immune regulation to joint homeostasis (**Figure 3B**) (Mann et al., 2024).

### 4.3 Other microbial metabolites

In addition to LPS and SCFAs, tryptophan and its derivatives also constitute crucial chemical mediators through which gut microbiota influence OA (Zhuang et al., 2023). Although specific regulatory pathways involving metabolites such as indoleacetic acid and kynurenine require further experimental verification, numerous cohort studies and preclinical experiments have widely indicated that disturbances in the tryptophan-indole pathway may initiate or exacerbate joint inflammation, pain, and matrix degradation (Binvignat et al., 2023; Wei et al., 2023a). Given the complexity and multiple dimensions of tryptophan metabolism, in-depth investigations into the upstream and downstream roles of gut microbiota in this metabolic pathway could potentially uncover novel molecular targets for OA therapy. Additionally, recent studies have reported a microbiota-mediated regulatory mechanism involving ferroptosis inhibition. The gut-derived metabolite capsiate suppresses HIF-1α expression and upregulates SLC2A1, thereby reducing chondrocyte apoptosis and inflammation induced by iron homeostasis imbalance (**Figure 3C**) (Guan et al., 2023).

### 4.4 Bone remodeling and subchondral bone change

Numerous recent studies have demonstrated that gut microbiota may be associated with processes, thereby affecting osteoarthritic bone phenotypes (Lu et al., 2021; Contino et al., 2022; Yang et al., 2024). One study revealed that germ-free mice subjected to non-invasive anterior cruciate ligament rupture, a model for PTOA, showed significantly reduced trabecular bone loss, suggesting that exogenous microbiota might serve as a catalyst linking acute inflammation with subsequent bone resorption (Hahn et al., 2021). Another study further employed microbiota transplantation experiments between “super-healer” MRL mice and OA susceptible mice, demonstrating that the microbiota from MRL mice protected recipient mice from severe cartilage degeneration and induced beneficial shifts in host immune phenotypes (Prinz et al., 2024). Collectively, these findings highlight the potential role of gut microbiota in maintaining osteoarticular integrity via immune and metabolic pathways. Recent studies have begun to elucidate the molecular mechanisms involved. In addition to immune modulation through microbial components such as LPS and SCFAs, gut microbiota also influences bone remodeling via endocrine signaling. Colonization with a healthy gut microbiota promotes the production of SCFAs, which in turn stimulate insulin-like growth factor-1 (IGF-1) synthesis in the liver and adipose tissue, may play a role in enhancing bone formation (**Figure 3D**) (Yan et al., 2018). Conversely, gut dysbiosis reduces SCFA availability, leading to lower IGF-1 levels and impaired skeletal development. SCFAs can also increase peripheral serotonin levels, which are thought to influence the differentiation and function of osteoblasts and osteoclasts. Disruption of this SCFA–IGF-1/serotonin axis has been implicated in bone abnormalities commonly observed in osteoarthritis. Incorporating this “microbiota–bone remodeling” relationship into the broader OA framework may help explain why microbial dysbiosis frequently coincides with osteophyte formation and subchondral bone sclerosis, thereby providing novel opportunities for the future development of microbiota-targeted interventions aimed at protecting osteoarticular structures (Zhu et al., 2024; Xi et al., 2025).

The mechanisms by which gut microbiota influence OA can be broadly summarized into four key aspects. First, transmembrane absorption of LPS induced by obesity and metabolic syndrome significantly exacerbates joint inflammatory responses (Guido et al., 2021); second, SCFAs function as critical immunomodulators, playing a central role in maintaining chondrocyte homeostasis and promoting autophagy (Kim, 2023); third, the tryptophan-indole and other microbial metabolites deeply participate in ferroptosis, oxidative stress, and chondrocyte metabolism (Yadav et al., 2024); Fourth, microorganisms have the ability to remotely regulate bone remodeling and subchondral bone changes, which can both enhance and protect joint structure (Zheng et al., 2024). Multiple studies have further validated, through obesity contexts and GWAS analyses, that specific microbial taxa may be associated with variation in inflammation and disease progression in OA. Approaches such as microbiota transplantation, probiotic administration, and microbial-derived metabolite supplementation have collectively provided novel perspectives for mechanistic research on OA, laying a solid foundation for future microbiota-targeted interventions in both clinical and animal studies (Schott et al., 2018; Yu et al., 2021). With the growing understanding of these mechanistic pathways, several therapeutic approaches targeting the gut microbiota have been proposed, including TCM.

## 5 Multi-target advantages and clinical potential of TCM

### 5.1 Monomeric extracts: quercetin and pterostilbene

Among monomeric extracts derived from TCM, quercetin and pterostilbene have attracted widespread attention due to their antioxidative and anti-inflammatory properties. In a study utilizing monosodium iodoacetate (MIA)-induced osteoarthritic rats, gavage for quercetin for 28 days significantly improved cartilage degradation and inflammatory markers, accompanied by beneficial changes in gut microbiota composition and associated metabolites such as SCFAs (Lan et al., 2021). These findings suggest that quercetin not only exerts direct anti-inflammatory and chondroprotective effects but also may help modulate disturbances in gut microbiota and associated metabolic pathways. Another study, conducted in mouse and *in vitro* models, confirmed that pterostilbene inhibited the activation of the NLRP3 inflammasome and NF-κB signaling pathways, thereby reducing chondrocyte apoptosis and joint damage. Importantly, pterostilbene was associated with a reduced abundance of inflammation-associated microbiota (Lee et al., 2024). These results indicate that monomeric extracts of TCM may exhibit dual therapeutic effects through integrated inflammatory control and microbial regulation in both joint and gut environments.

### 5.2 Regulation of microbiota and metabolic networks by TCM formulas

Regarding compound formulas, the multi-component nature typically imparts broader modulatory effects on both host and microbiota (Lin et al., 2021; Huang et al., 2022; Fan et al., 2023; Gou et al., 2023; Li et al., 2023; Jiang et al., 2024). One such example is Guizhi Shaoyao Zhimu Decoction (GSZD), a classical TCM formula from the Synopsis of Golden Chamber traditionally used to treat gout and rheumatoid arthritis, which comprises nine herbs including Cinnamomum cassia (Guizhi), Paeonia lactiflora (Shaoyao), and Anemarrhena asphodeloides (Zhimu). A study focusing on the effects of GSZD in a gouty arthritis model demonstrated that treatment markedly reduced joint swelling, bone erosion, and serum levels of uric acid and LPS (Bian et al., 2024). Concurrently, the abundances of beneficial gut bacteria, such as *Lactobacillus* and *Ruminococcaceae*, were increased, while pro-inflammatory bacteria including *Blautia* were reduced, thus suggesting a potential therapeutic pattern of “microbial correction combined with inflammation alleviation”. Similarly, Zushima tablet, commonly used in rheumatoid and osteoarthritis diseases also presents similar characteristics in rats with collagen-induced arthritis: it can not only improve joint pathology, but also may be involved in modulating host and bacterial metabolic pathways (Shan et al., 2018). Another study reported that Xiong Fu Powder reduced osteoclast accumulation and joint inflammation by restoring intestinal mucosal immunity and Treg/Th17 balance under conditions of spleen deficiency (Xi et al., 2022). Furthermore, Ershiwuwei Lvxue Pill was found to downregulate MMP expression and inhibit RANKL and NF-κB signaling pathways, while simultaneously optimizing the abundance of probiotic genera such as *Lactobacillus* and reducing arachidonic acid levels in the gut (Li et al., 2022). These findings illustrate that TCM formulas, through integrative effects of multiple active components, exert comprehensive therapeutic actions on arthritis via modulation of gut microbiota, inflammatory pathways, and energy metabolism (Xu et al., 2021; Zuo et al., 2023; Luo et al., 2024).

### 5.3 Moxibustion and electroacupuncture

In addition to oral administration of TCM, external treatments such as moxibustion also hold an essential position in traditional Chinese medical practice. One study applied moxibustion therapy to mice with ACLT-induced osteoarthritis by stimulating specific acupoints (Shenshu and Zusanli) continuously for 28 days, and results showed significant protective effects on cartilage and subchondral bone (Fu et al., 2023). Through 16S sequencing and untargeted metabolomics analysis, the intervention was confirmed to restore both gut microbiota composition and systemic inflammatory markers toward healthy levels. This beneficial effect may be mediated by modulation of critical pathways, such as the cAMP signaling pathway, providing novel insights into how localized stimulation can exert systemic effects through the gut–joint axis. Similarly, another study demonstrated that different courses of moxibustion treatment significantly alleviated cartilage damage and inflammation in a KOA rat model by reshaping gut microbiota balance, specifically by increasing beneficial bacterial populations and reducing pathogenic species, suggesting an association between microbiota modulation and improved OA symptoms (Jia et al., 2022). Similarly, electroacupuncture was found to regulate gut microbiota, mitigate inflammation, alleviate pain, and improve functional in KOA patients, thus opening a novel perspective for KOA interventions via the microbiota–inflammation axis (Wang et al., 2021).

TCM demonstrates potential multi-pathway advantages in modulating microbiota-host interactions. From monomeric extracts and herbal formulas to external treatments such as moxibustion, TCM can bridge immune-inflammation responses, gut microbial composition, and metabolic homeostasis (**Table 2**) (Hu et al., 2021; Wei et al., 2024). Compared with single-target Western medical approaches, TCM typically offers a holistic perspective with relative safety; however, rigorous, large-scale randomized controlled trials remain necessary to robustly evaluate clinical efficacy and potential side effects, as well as clarify specific molecular mechanisms through modern omics methodologies. Despite increasing use of omics-based approaches in this field, the current body of research remains largely correlative, lacking robust causal validation and mechanistic clarity. In addition, many studies suffer from methodological constraints, such as limited sample sizes, absence of longitudinal design, and inconsistent application of randomization and blinding procedures. These issues collectively hinder reproducibility and limit the generalizability of findings across populations and settings. Addressing these challenges will be essential to advancing TCM-based interventions beyond empirical observation toward evidence-based clinical application.

**Table 2:** Summary of studies on Traditional Chinese Medicine/natural product interventions in arthritis and gut microbiota regulation.

Treatment	Duration	Model		Subject	Mechanism	Result	Reference
Quercetin 100 mg/kg	8 weeks	Monoiodoacetate OA	rat		Reshaping the gut microbiota Altering gut microbiota metabolic products Modulating host metabolomics through the regulation of the gut microbiome	Increase the proportion of *lactobacilli* and elevate SCFAs levels Significant related changes are observed in host metabolomics	(Lan et al., 2021)
Pterostilbene 100 or 200 mg/kg	4 weeks	octacalcium phosphate induced OA	mice (n = 3)		Inhibition of the NLRP3 inflammasome and reduction of IL-1β and IL-6, suppressing NF-κB Alteration of the gut microbiota composition	Alleviate cartilage damage and inflammation levels Increase the proportion of *Bacteroidetes* and *Firmicutes*	(Lee et al., 2024)
Guizhi Shaoyao Zhimu Decoction 3.2, 1.6, 0.8 g/kg/d	3 weeks	Hyperuricemic diet combined with locally injected gouty arthritis induction	rat (n = 6)		Downregulation of TNF-α and IL-1β expression Inhibition of the TLR4 Inflammatory pathway improvement of purine metabolism Modulation of gut microbiota abundance	Serum uric acid and LPS levels decreased Joint swelling and bone erosion were reduced The abundance of *Lactobacillus* increased	(Bian et al., 2024)
Moxibustion 20min/d	4 weeks	ACLT OA	C57BL/6 mice (n = 6)		Regulation of immunity through the cAMP signaling pathway Restoration of the proportion of gut probiotics	Improve cartilage and subchondral bone structure Reduce systemic inflammation Validate the effect of external stimuli on the "gut-joint axis	(Fu et al., 2023)
Moxibustion	2 weeks, 4 weeks, 6 weeks	Monoiodoacetate OA	rat (n = 5, 6)		Regulate inflammatory factors Modulate gut microbiota abundance	Cartilage damage was significantly improved The abundance increased significantly after moxibustion Moxibustion only exerts a noticeable protective effect on knee joint cartilage when a certain cumulative dose is reached	(Jia et al., 2022)
Electroacupuncture	8 weeks	–	KOA population and healthy controls (n = 30)		Modulate the composition of the gut microbiota.	Electroacupuncture can also reverse bacteria associated with KOA, such as *Clostridium*, *Bacteroides*, *Agaricomycetes*, and *Streptococcus* OA pain was alleviated	(Wang et al., 2021)

Overall, Integration of TCM with probiotics, exercise, or other microbiota-targeted interventions may lead to a more comprehensive and personalized strategy for the prevention and treatment of OA and related joint inflammation. Beyond TCM, emerging interventions including probiotics, biomaterials, and dietary modulation have also demonstrated promising effects on the gut–joint axis.

## 6 Diversified microbial interventions: probiotics, innovative biomaterials, and nutritional regulation

### 6.1 Probiotics, prebiotics, and postbiotics

In OA prevention and treatment, a growing number of studies have focused on regulating gut microbiota to achieve anti-inflammatory effects and joint protection (Dunn and Jeffries, 2022; Liang et al., 2023; Gilat et al., 2025). One study demonstrated that administration of crude mulberry polysaccharides significantly ameliorated inflammation, cartilage degradation, and bone loss in a rat model of knee OA, while concurrently restoring a healthier gut microbiota composition (Zheng et al., 2024). When gut microbiota depletion or transplantation was performed in these rats, the protective effects of mulberry polysaccharides were correspondingly weakened or transferred, suggesting an association among polysaccharides, microbiota, and joint pathology. Conversely, another study showed that antibiotic-induced gut microbiota disruption in mice unexpectedly reduced knee inflammation and cartilage degradation to a certain extent, suggesting that extreme microbiota reshaping could occasionally confer experimental benefits (Guan et al., 2020). However, this observation also highlights concerns about safety and controllability, indicating that large-scale microbial disturbances might produce complex and unpredictable consequences. Thus, gentler regulatory methods such as probiotics, prebiotics, or postbiotics may provide greater clinical feasibility (Ji et al., 2023; Billington et al., 2023).

### 6.2 Low molecular weight chondroitin sulfate and collagen

Certain nutritional supplements have also gained attention in relation to OA and gut microbiota regulation (Ross et al., 202; Bhardwaj et al., 2021; Liu et al., 2023; Laurindo et al., 2024). Studies indicate that low-molecular-weight chondroitin sulfate extracted from sturgeon cartilage exhibits superior efficacy in OA treatment compared to conventional high-molecular-weight forms, manifesting in stronger anti-inflammatory effects and greater inhibition of joint cell apoptosis. Critically, these beneficial effects are directly associated with its ability to modulate gut microbiota composition, including increasing the abundance of beneficial bacteria such as *Akkermansia* and *Prevotellaceae_NK3B31_group*, and concurrently elevating short-chain fatty acid levels such as butyrate, thereby reducing inflammatory factors and matrix degradation associated with OA progression (Jing et al., 2025). In addition, studies have investigated the fermentative potential of collagen hydrolysates in the colon (Larder et al., 2021). Findings indicate that certain collagen supplements retain peptide structures accessible to gut microbiota following simulated gastrointestinal digestion *in vitro*, and are subsequently metabolized by intestinal microorganisms into short-chain fatty acids, branched-chain fatty acids, and other metabolites with prebiotic properties beneficial to host metabolism. By optimizing molecular weight and amino acid composition, both chondroitin sulfate and collagen peptides can directly support joint matrix homeostasis and simultaneously maintain or stimulate probiotic activity in the gut environment, thus presenting new opportunities for future interventions that integrate nutritional supplementation with microbiota regulation in OA management (Gentile and Weir, 2018; Yu et al., 2024).

### 6.3 Innovative biomaterial and conventional pharmaceutical

In addition to probiotics, prebiotics, and nutritional supplements, innovative biomaterials and conventional pharmaceuticals also exhibit potential in gut microbiota modulation (Lindell et al., 2022). Using GNPs in an ACLT-induced mouse model, researchers observed a significant attenuation of articular cartilage degeneration, an effect closely associated with modulation of the gut microbiota (Deng et al., 2024). Specifically, GNP treatment increased the abundance of beneficial bacterial taxa such as *Akkermansia* and *Lactobacillus*, enhanced the production of SCFAs including butyrate, and elevated levels of anti-inflammatory cytokines such as IL-10, collectively indicating a potential interaction mechanism involving the “nanoparticle–microbiota–joint” axis. Another study revealed that the conventional antihypertensive drug captopril not only reduced blood pressure in DOCA salt hypertensive rats but also decreased the abundance of pro-inflammatory gut bacteria, such as *Escherichia-Shigella*, consequently alleviating chondrocyte senescence and bone destruction (Chan et al., 2022). These findings suggest that contemporary or existing pharmaceuticals may also exert unexpected regulatory effects within the microbiota–joint axis. With appropriate therapeutic design and application, such strategies could synergistically enhance joint function (Garcia-Santamarina et al., 2024).

Whether using biological preparations such as prebiotics or probiotics, or employing low-molecular-weight chondroitin sulfate, collagen supplements, nanomaterials, or pharmaceutical combinations, these approaches collectively offer practical strategies for alleviating OA-associated inflammation, bone destruction, and pain through modulation of the gut microbiota. However, certain studies indicate that antibiotic-induced extreme dysbiosis can also temporarily relieve OA symptoms, suggesting that microbiota-targeted interventions might yield diverse experimental outcomes (Guan et al., 2020). Therefore, when translating these interventions into clinical practice, greater attention should be devoted to their safety and controllability.

Restoring or optimizing gut microbial homeostasis may help alleviate systemic inflammation and protect joint structures (Peng et al., 2021; Liu et al., 2023). Integrating probiotics or prebiotics, nanomaterials, and pharmaceuticals with individualized lifestyle interventions may establish a more comprehensive framework for OA management (Nicholson et al., 2012; Cheung et al., 2023). From supplementation with chondroitin sulfate and collagen peptides to the combined application of pharmaceuticals such as captopril, individually tailored strategies may yield improved therapeutic outcomes aligned with specific patient needs (Sun et al., 2023). With the deepening of subsequent trials and clinical studies, “diversified microbial intervention” may further enrich the frontier map of OA precision treatment. However, at the mechanistic level, although the association between SCFAs and macrophage polarization has been demonstrated, the specific molecular pathways and regulatory networks involving key bacterial strains remain poorly defined. In addition, the use of antibiotic treatment in experimental models does not fully replicate sterile conditions, which may compromise the reliability and interpretability of the results. In the context of translational medicine, important gaps persist, including a lack of well-designed dose–response studies and long-term safety assessments. Furthermore, the extrapolation of findings from animal models to clinical practice remains uncertain and requires further validation. These limitations underscore the need for more rigorous and mechanistically grounded investigations before microbiota-targeted strategies can be reliably implemented in OA management.

Collectively, these findings reinforce the therapeutic potential of targeting the gut microbiota in OA. Accordingly, the final section discusses overarching perspectives and future research directions.

## 7 Conclusion and perspective

With increasing life expectancy, age related conditions and diseases have become widespread and pose a significant social burden (Giovarelli et al., 2025). Osteoarthritis, a disease whose prevalence increases with age, is also receiving increasing attention (Steinmetz et al., 2023). In recent years, extensive evidence has confirmed the critical role of gut microbiota in the onset, progression, and symptomatic manifestations of OA (Ramasamy et al., 2021). Evidence ranging from epidemiological investigations to multi-omics analyses consistently indicates that microbial dysbiosis is closely associated with systemic inflammation and nutritional metabolic disturbances (Han et al., 2021; Bauermeister et al., 2022; Tansey et al., 2022; Takeuchi et al., 2023). These include endotoxin-induced low-grade inflammation, immune homeostasis mediated by short-chain fatty acids, and the effects of other microbial metabolites on chondrocyte ferroptosis (van Eeden et al., 2021; Bell et al., 2022; Zhao et al., 2022; Violi et al., 2023; Krause et al., 2024; Zhang et al., 2025). Additionally, gut microbiota can exert remote regulatory effects on bone remodeling, thereby either exacerbating or alleviating the pathological progression of OA (Ding et al., 2022; Wu et al., 2022; Aurora and Silva, 2023; Lyu et al., 2024).

The application of probiotics and prebiotics, the introduction of novel functional materials such as low-molecular-weight chondroitin sulfate and collagen peptides, integration of traditional therapies including Chinese traditional medicine and moxibustion, and microbiota-targeted interventions involving nanomaterials or conventional pharmaceuticals, have collectively demonstrated potential value for joint protection and inflammation alleviation. These approaches hold promise for establishing an integrated therapeutic model of “joint repair coupled with microbiota modulation”. However, most existing studies remain at the stage of animal experiments or small-scale clinical trials, while large-sample prospective randomized controlled trials are relatively limited. In real-world clinical practice, issues such as individual variability, the dynamic equilibrium of gut microbiota ecology, and long-term safety of interventions still require comprehensive investigation (**Tables 3**, **4**).

**Table 3:** Animal studies on the regulation of gut microbiota in osteoarthritis.

Intervention	Model	Duration	Subject	Mechanism	Results/Conclusion	Reference
cecal microbiota suspension of SPF mice	Aseptic model DMM OA model	4h, 24h, 48h, 1 weeks, 2 weeks, 4 weeks	GF mice (n = 6) C57BL/6J mice (n = 6)	Regulate the gut microbiota Modulate intestinal permeability	The microbial DNA in cartilage originates from the gut microbiota Serum LPS levels are elevated The proportion of *Actinobacteria*, *Staphylococcus*, and other bacteria increases	(Izda et al., 2023)
Fecal microbiota transplantation	Meniscal ligamentous injury OA	10 weeks	GF C57BL/6J mice	Affect systemic inflammation Impact gut microbiota abundance Increase intestinal wall permeability	Cartilage and synovial damage are aggravated The average systemic concentration of inflammatory biomarkers increases The abundance of *Ruminococcus* decreases	(Huang et al., 2020)
Microbiota transplantation	DMM OA	8 weeks	C57BL6/J mice (n = 9, 7)	Regulate systemic inflammatory factors Modulate gut microbiota composition	Osteophyte formation is reduced The proportion of protective bacteria such as *Lactobacillus* increases The microbiota may be a key mediator in the hormonal regulation of OA	(Schlupp et al., 2023)
Probiotics / Postbiotics	Sodium iodoacetate injection OA	24 days	Rat (n = 6)	Modulate gut microbiota metabolites Inhibit pathways such as NF-κB and NLRP3 Increase the expression of Foxp3	SCFAs production increases Joint damage and pain are alleviated Immune homeostasis is restored	(Cho et al., 2022)
Indole-3-propionic acid	ACLT OA	8 weeks	rat (n = 5)	Downregulate the expression of TNF-α and IL-1β Enhance chondrocyte activity Inhibit through the aryl hydrocarbon receptor/NF-κB	Cartilage degradation and synovial inflammation are reduced The expression of proteoglycans and type II collagen is significantly increased	(Zhuang et al., 2023)
Capsaicin, a metabolic product of the gut microbiota	DMM OA	8 weeks	mice (n = 9)	Capsaicin inhibits the expression of HIF-1α by activating SLC2A1	Ferroptosis slows the osteoarthritis progression	(Guan et al., 2023)
SCFA	Osteoclast-mediated arthritis	8 weeks	R26STAT3Cstopfl/flC mice	Inhibition of IFN in bone marrow progenitor cells affects osteoclast differentiation	The positive regulator of osteoclastogenesis, Car2, is downregulated Bone erosion ceases	(Yang et al., 2024)
Aseptic conditions	ACLT OA	1 week	GF mice (n = 8, 5) C57BL/6 mice (n = 10, 9)	Downregulate arachidonic acid metabolism, TCA cycle, arginine and proline metabolism, as well as pyruvate metabolism	The trabecular bone loss in GF mice is significantly lower The gut microbiota may promote the development of post-traumatic osteoarthritis (PTOA) during the acute phase by regulating the innate immune system	(Hahn et al., 2021)
Microbiota transplantation	DMM OA	8 weeks	C57BL6/J mice (n = 12) MRL/MpJ mice (n = 10, 6)	Modulate intestinal permeability Alter microbiota characteristics CD25+CD4+ T cells decrease	The gut microbiome is partly responsible for the OA protection in MRL mice This protection can be transferred through microbiota transplantation Transplantation induces systemic immune phenotype changes, which are associated with OA protection	(Prinz et al., 2024)
Oral Propolis Nanoemulsions	Ovariectomy-induced bone loss	8 weeks	mice (n = 5)	PNEs promote GM metabolite L-arginine to inhibit osteoclast formation and function	Bone resorption is significantly inhibited, while bone formation markers remain unchanged The trabecular structure is dense	(Zheng et al., 2024)
Oligofructose	DMM OA	12 weeks	mice (n = 6-8)	Modulate gut microbiota abundance Regulate serum inflammatory factors	The gut microbiota of obese mice is restored to a state similar to that of lean mice Cartilage damage and cartilage hypertrophy are alleviated	(Schott et al., 2018)
Crude mulberry polysaccharide	Sodium iodoacetate injection OA	4 weeks	SD rat (n = 9)	Regulate chondroitin sulfate Inhibit MMP-13 Modulate gut microbiota abundance Regulate bacterial activity through bacterial fermentation	Trabecular thickness, bone density, and bone volume fraction are improved α-diversity and β-diversity of the gut microbiota are increased Crude mulberry polysaccharides inhibit the progression of KOA by modulating gut microbiota composition, and their efficacy strictly depends on the presence of the microbiota	(Zheng et al., 2024)
Ampicillin (1.0 g) or Neomycin (0.5 g)	DMM OA	8 weeks	C57BL/6J mice (n = 9)	Reduce the expression of MMP-13 Lower serum calcium and increase serum magnesium Alter the composition of the gut microbiota	Trabecular thickness increases The levels of *Firmicutes* and *Bacteroidetes* are significantly lower in male mice compared to female mice	(Guan et al., 2020)
Milk-derived extracellular vesicles	DMM OA	10 weeks	C57BL/6J mice (n = 3, 5)	Modulate gut microbiota abundance	Cartilage damage is significantly alleviated The DMM-induced reduction of COL2A1 is largely reversed The abundance of *Gram-negative bacilli* decreases	(Liu et al., 2023)
low molecular weight chondroitin sulfate	Monoiodoacetate OA	8 weeks	C57BL/6 mice (n = 10)	Increase beneficial bacteria such as *Akkermansia* and *Prevotellaceae* Enhance butyrate production	Superior to the macromolecule CS It significantly improves both the joint matrix and gut microbiota	(Jing et al., 2025)
Gold nanoparticles	ACLT OA	8 weeks	C57BL/6J mice (n = 5, 7, 10)	Regulate the gut microbiota Enhance anti-inflammatory factors	Slow down cartilage degeneration Upregulation of *Akkermansia* and *Lactobacillus* produces a synergistic anti-inflammatory effect	(Deng et al., 2024)
Captopril	DOCA-salt induced hypertension	2 weeks	Rat (n = 8)	Inhibit the pro-inflammatory bacteria *Escherichia-Shigella* Reduce the accumulation of senescent cells in synovial joint tissues Lower the expression of MMP-13	Joint cartilage degeneration in hypertensive rats is improved, and DOCA-induced subchondral cell senescence is reversed Conventional antihypertensive drugs may protect cartilage through the gut microbiota	(Chan et al., 2022)

**Table 4:** Human studies on the regulation of gut microbiota in osteoarthritis.

Research	Sample size	Detection method	Finding	Conclusion	Reference
Identification of cartilage microbiome DNA characteristics and their correlation with knee and hip osteoarthritis	95	16s rRNA gene deep sequencing	Revealed microbial DNA markers in human cartilage	This characteristic changes during the development and progression of human OA, including an increase in *Gram-negative* components	(Dunn et al., 2020)
Changes in gut microbiota and metabolite profiles in patients with endemic osteoarthritis and Kashin-Beck disease in China	67	16S rDNA gene and metabolomic sequencing liquid chromatography-mass spectrometry (LC/MS).	In subjects with Kashin-Beck disease, the differential abundance of gut microbiota primarily belongs to the *Prevotella genus* Serum metabolomics analysis reveals that differential metabolites in grade I and II Kashin-Beck disease participate in lipid metabolism networks, including unsaturated fatty acids and glycerophospholipids	These differences in metabolite levels are associated with changes in the abundance of specific species These differences in metabolite levels are associated with changes in the abundance of specific species	(Wang et al., 2021)
Fecal metabolomics reveals the products of protein hydrolysis dysregulation and microbial metabolic changes in obesity-related osteoarthritis	92	Untargeted fecal metabolomics analysis 16S ribosomal RNA amplicon sequencing	Adults with obesity and knee and hand OA have distinct fecal metabolomes, characterized by increased protein hydrolysis products, leukotriene metabolism dysregulation, and changes in microbial metabolites	Metabolic dysregulation suggests a potential role of protein hydrolysis dysregulation in OA	(Rushing et al., 2021)
Diet influences knee osteoarthritis through gut microbiota and serum metabolites	200	16S rDNA sequencing Untargeted metabolomics study	The abundance of *Blautia* is enriched in the osteophyte group of KOA Serum metabolites LTB4 and PGD2 are expressed at higher levels in the KOA osteophyte group	KOA patients in Inner Mongolia, due to their preference for cheese, exhibit lower abundance of *Blautia* in the gut and reduced expression levels of five related serum metabolites, which may lead to a decrease in osteophyte formation	(Zhu et al., 2024)

In conclusion, gut microbiota research has expanded novel frontiers in the etiological understanding and therapeutic strategies for OA. Through continued advancement in comprehending microbiota-immune-metabolic interactions and actively integrating multidisciplinary approaches and advanced technological methodologies, significant breakthroughs are anticipated in the precise prevention and personalized treatment of OA, ultimately enhancing patient quality of life and overall health outcomes.
